# Supplementary material for: Coupling chemosensory array formation and localization
Source: eLife. 2017 Oct 23;6:e31058. doi: 10.7554/eLife.31058 (PMC5706961; doi:10.7554/eLife.31058)
Supplement: Supplementary file 1. — Table listing the strains and plasmids used in the study. [file elife-31058-supp1.docx]

**Table S1. Strain and plasmid list.**

| Strain name | Genotype | Reference |
| --- | --- | --- |
| *Vibrio cholerae* N16961 | Clinical isolate, wild type |  |
| *Vibrio cholerae* PM3 | *vc2060L209A (parPL209A)* | This work |
| *Vibrio cholerae* PM2 | *vc2060W305A (parPW305A)* | This work |
| *Vibrio cholerae* PM4 | *vc2060L209A-W305A (parP-L209A-W305A)* | This work |
| *Vibrio cholerae* SR70 | *Δvc2060 (ΔparP)* | This work |
| *Vibrio cholerae* AA13 | *Δvc2063 (ΔcheA1)* | This work |
| *Vibrio cholerae* AA15 | *Δvc2060 Δvc2063 (ΔparP ΔcheA1)* | This work |
| *Vibrio cholerae* AA17 | *vc2060L209A-W305A::vc2063 (parP-L209A-W305A ΔcheA1)* | This work |
| *Vibrio cholerae* AA21 | *Δvc2060::vc2060-ΔAIF Δvc2063 (ΔparP::parP- ΔAIF ΔcheA1)* | This work |
| *Vibrio cholerae* PM34 | *Δvc2060::vc2060-P5 Δvc2063 (ΔparP::parP-P5 ΔcheA1)* | This work |
| *Vibrio cholerae* PM35 | *Δvc2063::vc2063-ΔP5 Δvc2060 (cheA1-ΔP5 ΔparP)* | This work |
| *Vibrio cholerae* AA18 | *Δvc2063::vc2063-ΔP5 (ΔcheA1-ΔP5)* | This work |
| *Vibrio cholerae* *ΔparC* | *Δvc2061 (ΔparC)* | (Ringgaard *et al.*, 2011) |
| *Escherichia coli* DH5αλpir | *sup E44, ΔlacU169 (ΦlacZΔM15), recA1, endA1, hsdR17, thi-1, gyrA96, relA1*, λpir |  |
| *Escherichia coli* BTH101 | F- *cya-99 araD139 galE15 galK16 rpsL1* *hsdR2 mcrA1 mcrB1* | (Karimova *et al.*, 1998) |
| *Escherichia coli* SM10λpir | KmR, *thi-1*, *thr, leu, tonA, lacY, supE, recA*::RP4-2-Tc::Mu, λpir |  |
| *Escherichia coli* VS296 | *ΔcheR ΔcheB ΔcheW ΔcheA ΔcheY ΔcheZ Δtar Δtsr Δtap* | Victor Sourjik |
| Plasmid name | Relevant genotype / description | Reference |
| pCVD442 | Suicide vector for gene deletion | (Donnenberg and Kaper, 1991) |
| pMF390 | *P*BAD::*yfp* | (Yamaichi *et al.*, 2007) |
| pMF391 | *P*BAD::c*fp* | (Yamaichi *et al.*, 2007) |
| pUC19 | *Plac::* | (Norrander *et al.*, 1983) |
| pUT18C | *Plac::T18* | (Karimova *et al.*, 1998) |
| pKT25 | *Plac::T25* | (Karimova *et al.*, 1998) |
| pSR1033 | *P*BAD::*yfp-vc2059 (cheW1)* | (Ringgaard *et al.*, 2011) |
| pSR1102 | *P*BAD::*yfp-vc2060 (parP)* | (Ringgaard *et al.*, 2014) |
| pPM014 | *P*BAD::*yfp-vc2060W305A (parPW305A)* | This work |
| pPM015 | *P*BAD::*yfp-vc20602PM (parP2PM)* | This work |
| pAK2 | *Plac::T18-vc2060 (parP)* | This work |
| pAK7 | *Plac::T25-vc2059 (cheW1)* | This work |
| pAK8 | *Plac::T25-vc2060 (parP)* | This work |
| pAK9 | *Plac::T25-vc2061 (parC)* | This work |
| pAK10 | *Plac::T25-vc2063 (cheA1)* | This work |
| pAK14 | *P*BAD::*yfp-vc2063* | This work |
| pAK63 | *P*BAD::*yfp-vc2063-(P1-P4)* | This work |
| pAK72 | *P*BAD::*yfp-vc2063-P5* | This work |
| pAK84 | *Plac::T18-vca0068* | This work |
| pAK88 | *Plac::T18-vc1868* | This work |
| pAK86 | *Plac::T18-vca0658* | This work |
| pAK90 | *Plac::T18-vc1898* | This work |
| pAK105 | *P*BAD::*yfp-vc2060L209A (parPL209A)* | This work |
| pSR1218 | *Plac::T18-vc2060L196A (parPL196A)* | This work |
| pSR1219 | *Plac::T18-vc2060L209A (parPL209A)* | This work |
| pSR1220 | *Plac::T18-vc2060L212A (parPL212A)* | This work |
| pSR1221 | *Plac::T18-vc2060I215A (parPI215A)* | This work |
| pPM010 | *Plac::T18-vc2060W305A (parPW305A)* | This work |
| pPM011 | *Plac::T18-vc20602PM (parP2PM)* | This work |
| pAK80 | *Plac::T25-vc1898* | This work |
| pAA56 | *Plac::T18-vc1898L518R* | This work |
| pAA48 | *Plac::T18-vc1898L521R* | This work |
| pAA50 | *Plac::T18-vc1898N522R* | This work |
| pAA51 | *Plac::T18-vc1898A524R* | This work |
|  | *Plasmid for deletion of vc2060 (parP)* | This work |
| pAA60 | *P*BAD::*mCherry-vc1898* | This work |
| pAA74 | *Plac::mCherry-vc1898* | This work |
| pAA75 | *P*BAD::*cfp-vc2059 (cheW1)* | This work |
| pAA76 | *P*BAD::*yfp-vc20602PM-cfp-vc2059 (parP2PM, cheW1)* | This work |
| pAA77 | *P*BAD::*yfp-vc2060-cfp-vc2059 (parP, cheW1)* | This work |
| pAA78 | *P*BAD::*yfp-vc2060W305A-cfp-vc2059 (parPW305A, cheW1)* | This work |
| pAA79 | *P*BAD::*yfp-vc2060L209A-cfp-vc2059 (parPL209A, cheW1)* | This work |
| pAK13 | *Plasmid for deletion of vc2063 (cheA1)* | This work |
| pPM021 | *Plasmid for insertion of vc2060L209A (parPL209A) on the chromosome replacing the native vc2060 locus* | This work |
| pPM020 | *Plasmid for insertion of vc2060W305A (parPW305A) on the chromosome replacing the native vc2060 locus* | This work |
| pPM027 | *Plasmid for insertion of vc20602PM (parP2PM) on the chromosome replacing the native vc2060 locus* | This work |
| pAA44 | *Plasmid for insertion of vc2060-P5 (parP-P5) on the chromosome replacing the native vc2060 locus* | This work |
| pAA43 | *Plasmid for deletion of vc2063-P5 (cheA1-P5)* | This work |
|  |  |  |
