# Supplementary material for: Coupling chemosensory array formation and localization
Source: eLife. 2017 Oct 23;6:e31058. doi: 10.7554/eLife.31058 (PMC5706961; doi:10.7554/eLife.31058)
Supplement: Supplementary file 2. — Table listing the primer names and sequences used in the study. [file elife-31058-supp2.docx]

**Table S2. Primer list**

| Primer name | Primer sequence |  |
| --- | --- | --- |
| VC2063-BTH-CW | ccccctctagaggcagctatgagctacgaattagacgaagac |  |
| VC2063-BTH-CCW | cccccggtaccgcggccgctcagacgcgagaagcagctg |  |
| VC2059-BTH-CW | cccccggatccggcagctatgtcggaaacgactcagagag |  |
| VC2059-BTH-CCW | cccccggtaccgcggccgcttacatatgagccatttcgtccca |  |
| VC2060-BTH-CW | cccccggatccggcagctatgagtagtgcattgatatccagc |  |
| VC2060-BTH-CCW | cccccgaattcgcggccgcttaattttccagtgcttttacatcaag |  |
| VC2061-BTH-CW | cccccggatccggcagctatgatcgtttggagtgtagcaaacc |  |
| VC2061-BTH-CCW | cccccggtaccgcggccgcctactcatcgatagcgagcctc |  |
| VC1898-CW2 | ccccctctagaggcagctatggaaaccgcatcggagtcagg |  |
| VC1898-CCW1 | ccccggtaccgcggccgcttagagtttaaacctatggactaactc |  |
| VCA0068-CW2 | cccccggatccggcagctatgggcgcagcacaacaagcca |  |
| VCA0068-CCW1 | cccccggtaccgcggccgcttacacgcgaaaatactggagctg |  |
| VC1868-CW2 | cccccggatccggcagctatggttattagtgctgcgattggctct |  |
| VC1868-CCW1 | cccccggtaccgcggccgcctacaacgtaaagcgtcggcaat |  |
| VCA0658-CW2 | cccccggatccggcagctatgaatgtcacccatcagaatttacag |  |
| VCA0658-CCW1 | cccccggtaccgcggccgcctatttttgtgcaaactgcttcga |  |
| VC2063del-a | Ccccctctagatctgccaataaccctatgtttaaag |  |
| VC2063del-b | Ttcgtagctcatacgttacccct |  |
| VC2063del-c | aggggtaacgtatgagctacgaatctcgcgtctgattagctgcac |  |
| VC2063del-d | Ccccctctagatttcccaaaggtgaggtagctg |  |
| VC2060-L196A-CW | gatccgtgaaaaagatttccaagtcgcttattttgatgtcaatggtgtcac |  |
| VC2060-L196A-CCW | gtgacaccattgacatcaaaataagcgacttggaaatctttttcacggatc |  |
| VC2060-L209A-CW | caatggtgtcactttcgcagtgccagctgatgagttaggtgggattcatcg |  |
| VC2060-L209A-CCW | cgatgaatcccacctaactcatcagctggcactgcgaaagtgacaccattg |  |
| VC2060-L212A-CW | ctttcgcagtgccactcgatgaggcaggtgggattcatcgtatgacc |  |
| VC2060-L212A-CCW | ggtcatacgatgaatcccacctgcctcatcgagtggcactgcgaaag |  |
| VC2060-I215A-CW | gccactcgatgagttaggtggggctcatcgtatgaccacacttaac |  |
| VC2060-I215A-CCW | gttaagtgtggtcatacgatgagccccacctaactcatcgagtggc |  |
| VC2060-W305-cw | gcgtgagcaagtcggtaaacggcctgcgctcgcgggcatggtgaaagaaaaa |  |
| VC2060-W305-ccw | tttttctttcaccatgcccgcgagcgcaggccgtttaccgacttgctcacgc |  |
| VC1898-L518R-cw | caatattgctgagcagaccaaccgtttggcgctcaatgctgcgattga |  |
| VC1898-L518R-ccw | tcaatcgcagcattgagcgccaaacggttggtctgctcagcaatattg |  |
| VC1898-L521R-cw | ctgagcagaccaaccttttggcgcgcaatgctgcgattgaagcggc |  |
| VC1898-L521R-ccw | gccgcttcaatcgcagcattgcgcgccaaaaggttggtctgctcag |  |
| VC1898-N522R-cw | gagcagaccaaccttttggcgctccgtgctgcgattgaagcggcgcgtg |  |
| VC1898-N522R-ccw | cacgcgccgcttcaatcgcagcacggagcgccaaaaggttggtctgctc |  |
| VC1898-A524R-cw | accaaccttttggcgctcaatgctcggattgaagcggcgcgtgcaggag |  |
| VC1898-A524R-ccw | ctcctgcacgcgccgcttcaatccgagcattgagcgccaaaaggttggt |  |
| VC2060-PM-ins-a | Ccccccccgggatccctgcccatatgtctcttg |  |
| VC2060-PM-ins-b | Gctggatatcaatgcactactcat |  |
| VC2060-PM-ins-c | Atgagtagtgcattgatatccagc |  |
| VC2060-PM-ins-d | Ttaattttccagtgcttttacatcaag |  |
| VC2060-PM-ins-e | Cttgatgtaaaagcactggaaaattaa |  |
| VC2060-PM-ins-f | Ccccccccgggaaccgaccctcctgcgattac |  |
| VC2060-1-cw | ccccgagctctgtacaagatgagtagtgcattgatatccagc |  |
| VC2060-1-ccw | Ccccgtcgacttaattttccagtgcttttacatcaag |  |
| VC2060_CheWlike-c | Caagtcctctattttgatgtca |  |
| VC2060_CheWlike-d | Catagctatcaatgcttgaaca |  |
| VC2060_ CheWlike-XbaI-a1 | Ccccctctagaatatccagcgaacaagcgctg |  |
| VC2060-VC2063-b1 | cgcgacgccaaccatcaagaaatctttttcacggatcgt |  |
| VC2063_P5-c1 | ttgatggttggcgtcgcg |  |
| VC2063_P5-d1 | ctgcttgagcagatccgg |  |
| VC2060-VC2063-e1 | ccggatctgctcaagcagcttaatgcagggcttgatgta |  |
| VC2060_CheWlike-XbaI–f1 | Ccccctctagatttcgtcccattcttcatcgg |  |
| VC2063-1-cw | ccccctctagagacatcctcgagctcatgagctacgaattagacgaagac |  |
| VC2063-1-ccw | Cccccgcatgctcagacgcgagaagcagctg |  |
| VC2063-7-ccw | cccccgcatgctcaggtagagtcaatatcgatcgagc |  |
| VC2063-8-cw | ccccctctagagacatcctcgagctcctggctattcttcctaccttgat |  |
| Vc1898-cw | cccccgcatgcctagccgttcatcggattagagt |  |
| Vc1898-ccw | cccccgcatgcctagccgttcatcggattagagt |  |
| Vc1898-cherry-2-cw | cccccgcatgcatggtgagcaagggcgaggaggat |  |
| Vc1898-XmaI-ccw | ccccccccgggctagccgttcatcggattagagt |  |
| CFP-VC2059-cw | ccccctgtacagggtgtcggaaacgactca |  |
| CFP-VC2059-ccw | cccccgcatgcttacatatgagccatttcgtccca |  |
| ShDo-Spc-CFP-CheW | cccccgtcgactaaggaggatttataaagatggtgagcaagggcgagga |  |
|  |  |  |
